# The interplay of technology, family, and identity: Chinese adolescents’ self-presentation on Douyin

**DOI:** 10.3389/fpsyg.2025.1544224

**Published:** 2025-03-26

**Authors:** Qimin Zhang

**Affiliations:** London School of Economics and Political Science, London, United Kingdom

**Keywords:** self-presentation, TikTok (Douyin in China), digital behavior, parental mediation, adolescents

## Abstract

In the context of digitization and globalization, social media platforms, particularly TikTok, have become central spaces for adolescents’ self-presentation and social interactions. While TikTok has gained global popularity, its Chinese counterpart, Douyin, serves a similar role but within a distinct regulatory and cultural framework. This study investigated how Chinese adolescents constructed their digital identities on Douyin and examined the role of parents in this process. By integrating Erving Goffman’s self-presentation theory and Albert Bandura’s social learning theory, the research considered perspectives from adolescents and their parents across diverse socioeconomic backgrounds in Shanxi Province, China. Through in-depth qualitative interviews, it captured individuals’ psychological experiences and behavioral patterns. Thematic analysis revealed that adolescents’ digital behaviors are driven by psychological demands such as belonging, self-identity, and social recognition. The findings demonstrated a complex interplay between adolescents’ “front-stage” performances and “back-stage” behaviors on Douyin. Furthermore, parenting styles—whether restrictive or directive—significantly influenced adolescents’ behavioral and psychological adaptation. Adolescents developed multifaceted perceptions of their identities through observation, imitation, and content creation, affected by both the global digital landscape and local cultures. Parental mediation either fostered adolescents’ psychological security or undermined their autonomy when overly restrictive. Overall, this study uncovers the psychological complexities of adolescents’ digital identity construction and emphasizes the interplay among family, culture, and technology. By extending Goffman’s and Bandura’s theories to the Chinese cultural context, it offers valuable insights into adolescents’ psychological development and digital engagement.

## 1 Introduction

In the digital age, the rapid development of social media platforms has tremendously impacted teenagers’ social interactions and identity-construction processes. While TikTok has gained global prominence, its Chinese counterpart, Douyin, serves as a localized version tailored to the domestic market. Both platforms provide adolescents with engaging digital spaces that integrate self-expression, social interaction, and entertainment. TikTok’s (2021) global active user base had surpassed 1 billion, providing teenagers with a digital space that integrates expression, interaction, and exploration. Unlike social platforms such as Instagram which often emphasize idealized images, TikTok and Douyin allow adolescents to showcase their interests and explore their identities with their relaxed creative expression and authentic display of everyday life (Jerasa and Boffone, 2021). By creating short videos, participating in online challenges, and engaging with both local and global users, adolescents fulfill the psychological demand for a sense of belonging and identity while gradually expanding the boundaries of their self-perception.

Adolescent identity construction and psychological development do not occur in isolation but are nested within a multilevel social-ecological system. According to Bronfenbrenner’s (1992) ecological systems theory, adolescent development is multidimensionally influenced by microsystems (e.g., family and school), and macrosystems (e.g., culture and social norms). Within this framework, the family is a crucial element of the microsystem and significantly impacts adolescents’ digital behavior and psychological adaptation. Parenting styles, in particular, have a profound effect on adolescents’ satisfaction of psychological demands and behavioral patterns. For example, authoritative parenting styles help adolescents balance autonomous exploration with a sense of rules through a combination of high expectations and high support (Baumrind, 1978; Maccoby, 1992). Authoritarian parenting styles may lead to excessive conformity in adolescents’ behavioral choices, whereas permissive parenting styles may result in behaviors that lack clear boundaries (Baumrind, 1978; Spera, 2005).

China’s unique cultural context further empowers special connotations to family parenting styles. Influenced deeply by Confucian values, Chinese families prioritize educational achievement, family responsibility, and intergenerational connections (Peterson et al., 2005). These cultural traditions amplify the parental role in guiding and supervising their adolescents’ digital behaviors. However, with the popularization of digital technology, this guidance and supervision may also become a potential source of tension in the parent–child relationship. The conflict between individual autonomy and societal expectations has become particularly evident in adolescent social media use. The key question to comprehend this phenomenon is how the family parenting style affects adolescents’ identity construction and psychological requirements satisfaction in digital environments. Douyin, as China’s leading short-video platform, serves as a critical space where Chinese adolescents construct their identities at the intersection of global digital culture and local traditions. Given the distinctive sociocultural context of Chinese adolescents, this study seeks to explore their psychological needs and adjustment processes in digital environments, as well as how family and cultural contexts influence these complexities.

## 2 Literature review

This study utilizes Erving Goffman’s self-presentation theory and Albert Bandura’s social learning theory to examine the media behavior and psychological development of adolescents, with a particular emphasis on how Chinese adolescents construct their digital identities and the impact of their family environment. In this study, “identity” and “self” are regarded as interchangeable concepts and analyzed through external and internal factors.

### 2.1 Internal factors: self-presentation and psychological demand

Goffman’s (1959) theory of self-presentation provides valuable insights for understanding adolescents’ behavior on digital platforms. He likened the daily activities of an individual to a theater performance, distinguishing between two spaces: frontstage and backstage. On the front stage, individuals create an image that conforms to social expectations through selective expression and behavioral control. While the backstage allows for a more genuine and unfiltered presentation of oneself. Although the theory was originally used in face-to-face interaction scenarios, it remains relevant in the digital age. On digital platforms, this “frontstage-backstage” distinction becomes more complex. Digital platforms grant adolescents greater control over their self-presentation. They engage in impression management and selective sharing to meet social expectations or to portray an idealized self (Hogan, 2010; Higgins, 1987). For instance, TikTok and its Chinese counterpart, Douyin, offer various authoring tools that enhance creative self-expression through filters and soundtracks, while privacy settings enable users to control the visibility of their content, striking a balance between public and private domains (Hernández-Serrano et al., 2022; TikTok, 2024). However, platform-specific engagement norms also shape how users construct their digital identities. As Zeng et al. (2021) note, TikTok’s global expansion has cultivated distinct participation patterns, influencing how users curate content and interact within platform-defined constraints.

Beyond technological influences, socio-cultural contexts also play a crucial role in adolescents’ self-presentation. Similar to how Black girls use TikTok to create a “homespace” for self-expression and social belonging (Martinez, 2022), Douyin users strategically adjust their self-presentation to balance social visibility and authenticity. Peers further reinforce these self-presentation strategies. Davis (2014) noted that peers play a crucial role in adolescents’ online identity construction, affecting their behavioral choices and psychological adaptations. While social media promotes the construction of idealized images, adolescents gradually become more concerned with the consistency between their online and offline identities, highlighting the importance of authenticity (Back et al., 2010; Mascheroni et al., 2015; Davis, 2014). Bandura’s (1977) social learning theory further extends this perspective by stating that in social media environments, adolescents learn by observing the behavior of others, imitating trending content, and evaluating feedback. However, the algorithms of digital platforms also play a significant role in this process. Brady et al. (2023) state that algorithmic recommender systems influence the information individuals are exposed to and the way it is presented, adding a non-human dimension to social learning. This algorithm-driven social learning not only alters adolescents’ behavioral patterns but also impacts their psychological adjustment and identity construction.

### 2.2 External factors: the impact of technology domestication and family mediation

Domestication Theory provides an insightful framework for understanding how families adopt and assign meaning to technology (Silverstone et al., 1992). The theory proposes that the integration of technology is divided into four stages: appropriation, objectification, incorporation, and conversion. These stages illustrate how technology evolves from being introduced externally to becoming an integral part of daily life, emphasizing not just its usage but also its significance as a symbol of culture and identity (Morley, 2003). Instead of passively accepting technology, family members actively reshape it as part of their lives through daily practices (Bakardjieva, 2006). Adolescents, as part of their family’s technological environment, are directly and indirectly influenced by their parents’ media use behaviors. The family serves as a crucial site for technology domestication, and its cultural and social dynamics deeply affect how technology is perceived and used. In the integration stage of domestication theory, parents monitor and intervene to change their teenagers’ media consumption, creating family interaction patterns (Haddon, 2018). This process is supported by a refined framework of parental mediation strategies. Valkenburg et al. (1999) categorized three types of parental mediation strategies: restrictive mediation, instructive mediation, and social co-viewing, corresponding to different levels of involvement and intervention. With the proliferation of mobile technology and social media, traditional mediation strategies have evolved toward more interactive models such as “active co-use” (Livingstone and Helsper, 2008), which encourage parents and adolescents to explore digital media together, fostering trust and media literacy.

However, there are significant differences in the selection and implementation of mediating strategies across cultures. As described by Haddon (2006), China’s one-child policy has resulted in a family environment that is more focused on individual development, and parents may support academic tasks by limiting recreational activities like television viewing. By integrating the theories of technology domestication and parental mediation, a more comprehensive understanding of adolescents’ digital behaviors can be achieved. Douyin’s recommendation algorithms and interaction mechanisms provide unique scenarios for adolescents’ self-exploration and identity construction. Meanwhile, the family’s cultural context and parental mediation strategies play a crucial role in shaping this exploration process.

This study investigates the family environment’s effects on adolescents’ digital behaviors by integrating theories of technology domestication and parental mediation strategies. It also employs Goffman’s self-presentation theory and Bandura’s social learning theory to analyze how adolescents construct their identities on Douyin and address their psychological demands. The core research questions of this study include: Based on Goffman’s self-presentation theory, how can we assess adolescents’ identity construction on Douyin concerning their psychological demands? How do the technological features of Douyin impact adolescents’ self-presentation? In what ways does this influence affect their psychological adjustment? Within the family environment, how do parents balance the tension between adolescents’ independence and their dependence on digital platforms through various strategies? By exploring these questions, this study aims to enhance understanding of adolescents’ media behaviors and psychological development, particularly within the context of Chinese traditional culture.

## 3 Research design and methodology

### 3.1 Research design

This study aims to explore in depth the self-presentation behavior of Chinese adolescents on the Douyin platform and the influence of their family environment. A qualitative research method was employed, with data collected through semi-structured interviews and analyzed thematically. The interview methodology was based on constructivist epistemology, emphasizing participants as active meaning constructors to tap into their psychological experiences and social behaviors in a digital context (Warren, 2002; DiCicco-Bloom and Crabtree, 2006). The flexibility of semi-structured interviews supports the exploration of complex social phenomena (Brennen, 2017). This study combined the strengths of both structured and unstructured interviews by establishing a framework of main topics while allowing for adjustments in the order of questions or the exploration of new topics based on interviewee responses. Such dynamic adjustment is particularly crucial for understanding adolescent digital behavior and the cultural and familial factors behind it. The one-on-one interview format further enhances the depth and privacy of the study by ensuring that respondents are able to express themselves freely in a safe environment (Kaplowitz, 2000; Elmir et al., 2011). In addition, video elicitation techniques were introduced into the interviews to encourage respondents to discuss Douyin videos that they considered important or typical, providing context-specific behavioral and psychological feedback (Liebenberg et al., 2013).

### 3.2 Data collection and analysis

The study employed purposive sampling, selecting six pairs of parents and adolescents from three middle schools in Shanxi Province, China. This sampling strategy ensured diversity and representativeness across different socioeconomic backgrounds and parenting styles, including authoritative, authoritarian, and permissive approaches. Given that parenting styles moderate the effectiveness of different mediation strategies (Ren and Zhu, 2022), this study also examined how authoritative, authoritarian, and permissive parenting styles influenced parents’ digital mediation practices. Although the sample was confined to Shanxi Province, this choice provided the study with the opportunity to explore local cultural and family influences in depth. All adolescent participants owned personal smartphones and independent Douyin accounts, which facilitated their digital engagement and autonomy. Data collection adhered strictly to standardized procedures to ensure reliability and authenticity. All interviews were conducted remotely via Tencent Meeting to maintain safety and convenience. Before the interviews began, the research team provided detailed instructions to both underage participants and their guardians, highlighting data anonymity and privacy protection measures. To enhance the quality and depth of the interview data, the study refined the interview guide based on feedback from a pilot study. This involved adjusting the wording and openness of questions to avoid leading participants, allowing them to express themselves freely. Additionally, video elicitation techniques were incorporated into the interviews, where participants were asked to reflect on their Douyin usage by discussing specific videos they had created, interacted with, or found influential. This approach provided enhanced perspectives into their self-presentation strategies and content management decisions. The data were analyzed using thematic analysis with Nvivo 14 software, employing a combination of deductive and inductive coding. Deductive coding drew on established theoretical frameworks, such as “ideal self” and “parental mediation strategies,” while inductive coding generated emergent themes, including “algorithm-driven behavioral patterns” and “adolescents’ re-domestication strategies.” This dual coding approach reinforced the applicability of existing theories while uncovering new insights into adolescents’ digital behaviors (see Table 1).

**Table 1:** Demographic information about the interviewees.

Pseudonym	Gender	Grade	Parent pseudonym	Relationship	Parental Style
Tina	Female	12	Olivia	Mother	Permissive
Zhang	Male	11	Sophia	Mother	Authoritarian
Charlie	Male	12	Jim	Father	Authoritative
Xing	Female	10	Emily	Mother	Authoritative
Rong	Female	12	Charlotte	Mother	Authoritarian
Wang	Male	11	James	Father	Authoritative

### 3.3 Ethical considerations

This study adhered strictly to ethical guidelines to protect participants’ rights and privacy. Written informed consent was obtained from all participants, and minors required guardian approval to participate. Data collection and storage were fully anonymized, with personal information deleted immediately after transcription. Additionally, all data were securely stored on a password-protected and encrypted platform to ensure confidentiality.

## 4 Results and discussion

### 4.1 Psychological demand: adolescents’ identity construction in Douyin

Adolescents’ identity construction on Douyin reflects their multifaceted psychological demands, including exploration, learning, belonging, and self-expression. As a digital space, Douyin not only facilitates knowledge acquisition and social interaction but also serves as a critical platform for negotiating identities at the intersection of global and local cultures. Through engagement with diverse content, adolescents enhance their self-efficacy, aligning with Bandura’s (1977) social learning theory, which posits that observation and imitation facilitate competence acquisition. For instance, Rong shared her preference for global pop culture, particularly K-pop videos, which reinforced her cultural identity and strengthened her connection to the global community. In contrast, Xing expressed enthusiasm for hanfu (traditional Chinese clothing) and handicrafts, emphasizing that these interests deepened her understanding of history and culture while allowing her to support local traditions through content creation. Such behaviors exemplify Hall’s (1997) theory of cultural identity, where dynamic cultural exploration helps adolescents shape and confirm their self-identity.

Zulli and Zulli (2020) highlight that TikTok fosters “imitation publics,” where identity construction is shaped through platform-driven mimetic participation rather than purely individual expression. The platform’s algorithm encourages engagement with pre-existing formats, reinforcing structured replication in self-presentation. This algorithmic influence extends to how adolescents build social connections on Douyin. Analyses revealed that adolescents actively engaged with their real-life social circles and expanded their interest-based virtual communities through platform interactions. Charlie and Wang reported that they maintained close relationships with friends by liking, commenting on, and sharing videos. These activities reflect the concept of “connectivity” (Treem and Leonardi, 2016), where instantaneous interaction fosters relational ties. Additionally, Rong shared her experience of finding online friends with similar interests through Douyin’s algorithmic recommendations, which significantly broadened her social network. The platform’s group functions also facilitate close social bonds centered on shared interests, enhancing group belongingness. As Madianou’s (2016) concept of “ambient co-presence” suggests, even simple acts like liking or sharing content contribute to sustaining emotional connections, offering new avenues for modern social identity construction. However, this persistent social connectivity also has potential drawbacks. While ambient co-presence fosters social bonds, it can simultaneously create a heightened sense of self-surveillance, where adolescents feel continuously observed and evaluated—even in asynchronous interactions (Madianou, 2016). Rong, for instance, reported experiencing anxiety about maintaining their online presence, fearing that a lack of engagement could lead to social exclusion. This demonstrates that while social media platforms like Douyin facilitate belonging, they can also amplify social pressures and emotional strain.

On Douyin, adolescents’ expression of diverse identities focuses on the dynamic management of ideal, actual, and real selves. Papacharissi (2010) and Papacharissi (2013) suggests that social media platforms provide individuals with an arena for self-presentation and identity management. On digital media platforms, adolescents use multimedia tools such as text, images, and video to perform themselves, forming their identities through constant reflection and association with social circles (Papacharissi, 2013, p. 208). Zhang and Xing described using curated content to showcase desirable traits such as artistic creativity and athletic prowess, aligning with Goffman’s (1959) dramaturgical metaphor of front-stage performances designed to meet audience expectations. Simultaneously, adolescents present their actual selves through unedited footage of their daily lives, demonstrating a need for authenticity. Tina noted that Douyin’s anonymity features and facial effects tools provide a safe space for her to explore her true self, enabling her to balance public and private expression while safeguarding privacy. However, even when adolescents seek authenticity, they remain aware of how their content is perceived and adjusted accordingly. As Ji (2024) highlights, social media platforms introduce context collapse, where adolescents must manage self-presentation across multiple audiences, including family, peers, and broader online communities. This forces them to engage in strategic self-presentation, carefully curating content to navigate different social expectations and minimize potential conflicts. Gran (2025) further argues that self-presentation on social media is not a rigid separation between frontstage and backstage but rather a continuous negotiation. The “Not-Not-Me” approach highlights how users strategically balance idealization and authenticity under audience expectations. This strategic balance is evident in how adolescents adjust their self-presentation not only to meet social expectations but also to align with platform affordances and audience responses.

Social feedback plays an important role in adolescents’ identity construction. Interactive behaviors such as liking, commenting, and sharing become key ways for them to acknowledge and reflect on their performance (Cipolletta et al., 2020; Baumeister and Hutton, 1987). Zhang and Xing illustrated that the diversity of social feedback shaped their motivations for self-expression, with positive feedback reinforcing their self-identity and boosting their social standing, whereas negative feedback may induce social anxiety. Zuo and Wang’s (2019) study further suggests that adolescents’ interactions on Douyin are not only superficial exchanges but also a deep process of constructing social recognition and identity belonging. In summary, Douyin fulfills adolescents’ psychological needs by facilitating learning, social interaction, and self-expression. While it enhances self-efficacy, belonging, and identity construction, its persistent social connectivity may also heighten self-surveillance and social pressures, requiring adolescents to navigate their online identity strategically.

### 4.2 Psychological adjustment: functions of Douyin and adolescents’ psychological balance

Adolescents’ psychological adjustment on Douyin is mainly characterized by peer-driven platform preferences, privacy protection, and self-management, as well as strategic behaviors to cope with algorithmic influences. These behaviors reflect their ability to dynamically adjust their self-expression and adapt to technological challenges in the digital environment. In terms of privacy protection and self-management, adolescents balance self-expression and privacy needs through back-stage behaviors and information control (Goffman, 1959). They selectively share personal information, keeping their private lives and emotional experiences limited to trusted circles. For instance, Rong and Wang mentioned that they restrict sensitive content, such as daily activities and private emotions, to close friends while focusing public posts on neutral topics like birthday celebrations or holiday greetings. This selective sharing aligns with Treem and Leonardi’s (2016) concept of “visibility” affordances, where users control the public scope of information to manage their identity and interactions effectively. Further analysis revealed that adolescents demonstrate high levels of proactivity and reflexivity in information management. For example, Wang and Zhang shared that they sometimes hide or delete certain videos after a period to optimize their long-term online presence. This practice reflected Papacharissi’s (2013) theory of the online public arena, where individuals adapted to the networked public stage by continuously evolving their identity performances through reflection and interaction. The flexibility of these content management strategies enables adolescents to fulfill their social needs while maintaining personal boundaries, striking a balance between openness and privacy.

When confronted with algorithmic influences, adolescents show sensitivity and adaptability to platform dynamics. TikTok’s recommendation system continuously adapts to user engagement patterns, shaping content visibility and interaction dynamics (Miltsov, 2022). In response, some adolescents adjust their self-presentation strategies to align with perceived engagement trends. For example, Wang described deleting or hiding videos that underperformed in likes or views to maintain the appeal and freshness of their content. This strategic adjustment reflects their awareness of the content lifecycle and reveals how algorithmic data shapes their self-presentation. However, adolescents do not passively accept these pressures. Instead, they actively observe, imitate, and adapt their behaviors in a dynamic process (Huang and Miao, 2020). Although some participants reported occasional shifts to other platforms such as Kuaishou, most emphasized that their platform choice was primarily shaped by peer influence rather than functional differences. Since their friends and classmates predominantly used Douyin, they preferred to stay on the platform to maintain social connections. As Xing noted, “I use Douyin just because most of my friends are there.” This highlights the role of peer networks in shaping adolescents’ digital engagement. Comprehensively, Douyin, as a highly personalized and interactive platform, provides adolescents with a space for psychological adjustment in the digital environment. Through privacy protection and information control, they effectively manage self-expression and social interactions. By coping with algorithmic interference, they exhibit strategic behaviors and a high degree of flexibility. These practices not only alleviate psychological stress caused by digital platforms but also support their identity construction and psychological balance in the digital society.

### 4.3 Developmental conflict: parental mediation and adolescent Independence conflict

Parental mediation strategies play a significant role in shaping adolescents’ independence and digital behaviors. These strategies can be categorized into two main forms: restrictive interventions and instructive mediation, each exerting distinct effects on adolescents’ psychological development, social behaviors, and independence construction. The findings suggested that parenting styles influenced the way parents implemented these mediation strategies. Authoritarian parents tend to impose strict restrictions on Douyin use, whereas authoritative parents are more likely to engage in instructive mediation, guiding their adolescents through discussions rather than rigid enforcement. In contrast, permissive parents often adopt a more hands-off approach, allowing adolescents to manage their own media use with minimal intervention. These variations indicate that parenting style not only shapes parental mediation strategies but also plays a role in adolescent psychological adjustment.

Restrictive mediation involves strict controls over adolescents’ Douyin usage, including limiting screen time, prohibiting access to certain content, or enforcing usage restrictions during specific periods. Parents like Emily, Sophia, and Charlotte made it clear that they used coercive tactics to lower their teenagers’ Douyin activity frequency. Examples of these tactics included limiting daily usage hours or preventing their children’s access to entertainment videos or other content that was judged unsuitable for their age group. They also imposed restrictions on using Douyin during designated times, like study or family time. These approaches aim to mitigate online risks and safeguard academic performance. However, despite these efforts, almost all parents reported that they did not enforce Douyin’s “teenager mode.” Many found it impractical, as its content filtering system primarily recommended cartoons and child-oriented videos, which failed to align with the interests of middle and high school students. Consequently, most adolescents disabled the feature themselves, illustrating a discrepancy between platform-imposed safety measures and actual user behaviors. While restrictive mediation can enhance online safety, it often triggers resistance. Some adolescents expressed a heightened desire for autonomy and personal space in response to these constraints. To navigate these restrictions, some engaged in subtle self-presentation strategies, such as maintaining separate public and private accounts or adjusting their content to align with parental expectations while retaining a more authentic identity within peer circles. Mainardi and Krijnen (2023) found that Italian adolescent girls adopted similar tactics to manage parental oversight on social media, including selectively curating their online personas, using coded language, or migrating to less monitored platforms. Martinez (2022) further highlighted that Black girls on TikTok strategically adjusted content visibility and used private accounts, not only to circumvent parental surveillance but also to navigate broader social and algorithmic constraints while maintaining digital autonomy. These findings suggest that while restrictive mediation offers protection, it may also fuel creative resistance, prompting adolescents to develop multifaceted strategies to balance parental expectations with their personal digital agency. This paradox underscores the complexity of restrictive interventions. While they aim to create a safer online environment, they may simultaneously limit opportunities for self-expression and identity exploration on Douyin.

Instructive mediation has shown a more positive role in parenting compared to unilateral restrictions. This strategy involves collaborative rule-setting through consultation and discussion, rather than imposing rigid limits (Valkenburg et al., 1999). For example, parents like Jim and James described guiding their adolescents in selecting appropriate content and discussing platform risks, transitioning from direct oversight to indirect support as their children matured. This progression aligns with the “incorporation” and “conversion” phases of technology domestication theory (Silverstone et al., 1992), in which digital platforms become an integral part of family life. Beyond active mediation, many parents assumed the role of “digital gatekeepers,” selectively sharing Douyin content with their children to shape their perspectives and influence online behaviors. Some used Douyin as an educational tool, accessing parenting advice and forwarding relevant videos to their children as conversation starters. For instance, Emily not only monitored her child’s Douyin activity but also used the platform to access educational videos on parenting, which she later applied in daily family interactions. However, this approach was not always well received. Several adolescents expressed frustration that their parents’ content preferences—largely shaped by algorithmic recommendations—did not align with their own, creating a “filter bubble” that reinforced generational differences in digital consumption. While some parents viewed content-sharing as a way to bridge communication gaps, many adolescents perceived it as an imposition of parental values, leading to tensions rather than mutual understanding. Intergenerational conflicts also emerged in privacy management and time allocation. Adolescents sought the freedom to explore identities and express emotions on Douyin, while parents prioritized academic performance and online safety. This divergence in values often led to disagreements, reflecting broader tensions between adolescent autonomy and parental control. Despite these tensions, adolescents did not passively accept parental control. Many employed strategic negotiation techniques, selectively engaging with parental recommendations while maintaining independent peer-based interactions.

Ultimately, the findings suggest that while parental mediation strategies influence adolescents’ digital behaviors, their role in shaping identity construction is neither deterministic nor uniform. Identity construction is a dynamic process shaped by negotiation between parental expectations, peer influences, and platform affordances. While restrictive parental mediation may create tensions by limiting self-expression, instructive mediation fosters a more collaborative environment, allowing adolescents to integrate parental guidance with their own digital agency. These findings highlight the importance of understanding adolescent identity construction as a multifaceted process, in which family, social, and digital dynamics continuously interact.

## 5 Conclusion

Focusing on Chinese adolescents’ self-presentation and identity construction on the Douyin platform, this study integrates Goffman’s self-presentation theory and Bandura’s social learning theory to explore adolescents’ behavioral patterns and the influences of their families in a digital context. Through semi-structured interviews, the research revealed that adolescents utilized Douyin to balance self-expression, privacy protection, and social expectations. The findings highlight the significant role of family mediation in shaping how adolescents engage with social media. Adolescents’ self-presentation is shown to be a multidimensional and dynamically adjusted process. They navigate the tension between public and private personas through backstage behaviors and selective sharing, constructing and reshaping their identities online. Douyin’s features, such as visibility, algorithmic recommendations, and editorial flexibility, provide adolescents with a high degree of freedom on the platform and create an environment for social interaction. In this environment, adolescents not only satisfy their sense of belonging and identity needs through social interactions but also enhance their sense of self-efficacy by observing and imitating content creators. Families play a pivotal role in adolescents’ use of Douyin by monitoring and guiding their activities. While restrictive interventions can protect adolescents from potential risks, they may also hinder the development of autonomy. In contrast, instructive mediation fosters greater independence and responsibility in self-management through negotiation. The choice of intervention strategies is closely related to the family’s cultural context, especially in Chinese families that prioritize academic achievement. Additionally, the phenomenon of parents gaining parenting insights from social media and applying them in their family dynamics illustrates how technology has been domesticated in contemporary parenting practices.

Despite its contributions, this study has certain limitations. As a qualitative study focusing on individual experiences, it does not establish a direct causal relationship between parental mediation strategies and adolescent identity construction. While parental influence is significant, peer networks, platform affordances, and socio-cultural norms also shape digital identity formation. Additionally, this study is based on a limited sample from three secondary schools in Shanxi Province, which, while providing valuable insights, may not fully represent broader adolescent experiences. Future research should expand to different cultural and geographic contexts to examine variations in social media engagement. A comparative approach could also help explore how identity construction differs across digital ecosystems. Furthermore, while this study focuses on adolescents’ perspectives, future research should incorporate parental viewpoints to better understand intergenerational digital interactions. Mixed-methods approaches or large-scale studies could further explore how adolescents negotiate identity construction under different family mediation styles. Expanding this research will provide a deeper understanding of adolescents’ self-presentation strategies in the evolving digital landscape and contribute to the development of theoretical frameworks on identity formation in a globalized, digitalized world.

## Data Availability

The datasets presented in this article are not readily available due to Non-Disclosure Agreements (NDA). Requests to access the datasets should be directed to QZ, zhangqimin104@gmail.com.
